# Environment Characterization in Sorghum (*Sorghum bicolor* L.) by Modeling Water-Deficit and Heat Patterns in the Great Plains Region, United States

**DOI:** 10.3389/fpls.2022.768610

**Published:** 2022-03-03

**Authors:** Ana J. P. Carcedo, Laura Mayor, Paula Demarco, Geoffrey P. Morris, Jane Lingenfelser, Carlos D. Messina, Ignacio A. Ciampitti

**Affiliations:** ^1^Department of Agronomy, Kansas State University, Manhattan, KS, United States; ^2^Corteva Agriscience, Johnston, IA, United States; ^3^Department of Soil and Crop Science, Colorado State University, Fort Collins, CO, United States; ^4^Horticultural Sciences Department, University of Florida, Gainesville, FL, United States

**Keywords:** adaptation, simulation, stress, climate, drought

## Abstract

Environmental characterization for defining the target population of environments (TPE) is critical to improve the efficiency of breeding programs in crops, such as sorghum (*Sorghum bicolor* L.). The aim of this study was to characterize the spatial and temporal variation for a TPE for sorghum within the United States. APSIM-sorghum, included in the Agricultural Production Systems sIMulator software platform, was used to quantify water-deficit and heat patterns for 15 sites in the sorghum belt. Historical weather data (∼35 years) was used to identify water (WSP) and heat (HSP) stress patterns to develop water–heat clusters. Four WSPs were identified with large differences in the timing of onset, intensity, and duration of the stress. In the western region of Kansas, Oklahoma, and Texas, the most frequent WSP (∼35%) was stress during grain filling with late recovery. For northeast Kansas, WSP frequencies were more evenly distributed, suggesting large temporal variation. Three HSPs were defined, with the low HSP being most frequent (∼68%). Field data from Kansas State University sorghum hybrid yield performance trials (2006–2013 period, 6 hybrids, 10 sites, 46 site × year combinations) were classified into the previously defined WSP and HSP clusters. As the intensity of the environmental stress increased, there was a clear reduction on grain yield. Both simulated and observed yield data showed similar yield trends when the level of heat or water stressed increased. Field yield data clearly separated contrasting clusters for both water and heat patterns (with vs. without stress). Thus, the patterns were regrouped into four categories, which account for the observed genotype by environment interaction (GxE) and can be applied in a breeding program. A better definition of TPE to improve predictability of GxE could accelerate genetic gains and help bridge the gap between breeders, agronomists, and farmers.

## Introduction

Sorghum (*Sorghum bicolor* L.) crop improvement during the last six decades has been associated with targeted changes in genotype (G), management practices (M), and environment (E), with crop productivity considered as the outcome of a complex G x E x M interaction (Duvick and Cassman, 1999; Assefa and Staggenborg, 2010; Ciampitti et al., 2020). However, little is understood about the relative contribution of each component (G x E x M), and many uncertainties on the degree (i.e., magnitude in yield changes) or the direction (positive or negative changes) of the responses are still common for sorghum production. Therefore, tailoring the right combination of G x M to the right E is critical to increase productivity and reduce the impact of abiotic stressors (Garnett et al., 2013; Hammer et al., 2014, 2020). In the presence of G x E, the effectiveness of genotype evaluation is highly influenced by (i) the ability to discriminate genotypes within an environment, (ii) the representativeness of this environment within the target population of environments (TPE), and (iii) its repeatability (Yan et al., 2011).

The concept of TPE was first introduced by Comstock (1978) and defined as the set of “types” of environments that exist across seasons within the spatial region of spanning a breeding program. Subsequent interpretations of the original TPE concept considered it as a mixture of repeatable environment-types that discriminate germplasm (e.g., Cooper et al., 2005). For many TPEs, the typical sizes of multienvironment trials (METs) and the traditional site-year sampling strategy can result in an inadequate representation of some environments in TPE (Chapman et al., 2000, 2003). Therefore, breeding strategies designed to exploit components of genetic variation associated with GxE interactions need to characterize the TPE. Environments that discriminate would maximize genetic advance using limited resources of land, labor, time, and equipment for plant breeding (Hamblin et al., 1980). Thus, environmental characterization is a critical step in defining the TPE suitable for genotype evaluation upon the trait targets of the breeding program.

Since drought stress and heat are both ubiquitous in the western United States (US) (Raz-Yaseef et al., 2015; Steiner et al., 2018) and causal of GxE in multiple crops, including sorghum (Chapman et al., 2000), a TPE characterization for sorghum in the US will be most impactful in US sorghum breeding. The Great Plains region of the US, herein termed as the “Sorghum Belt,” accounts for three-fourth of the country-grain production (estimated at 9.4 × 10^6^ Mg in 2020), with most of the crop region distributed among the states of Kansas, Texas, Colorado, and Oklahoma (Ciampitti et al., 2019). This region is highly variable in both spatial–temporal scales due to contrasting soil types (soil texture, pH, depth, organic matter content; Soil Survey Staff et al., 2021), management systems, and interannual rainfall (west to east from more than 1,000 mm to less than 500 mm in an annual basis: Lin et al., 2017). Furthermore, in environments with high seasonal variability, where climatic rather than edaphic factors determine yield, the repeatability at the site is a major concern, highlighting the need for breeding sites where between-season variation is maximized and within-season variation is minimized (Goodchild and Boyd, 1975; Boyd et al., 1976). Moreover, in these environments, including a seasonal classification, for a better interpretation of the G x E interaction would allow to improve the genetic gain rate (Chenu et al., 2011; Demarco et al., 2021). Lastly, enhancing genetic gain *via* yield increases of sorghum in this region cannot only increase the stability of production under limited water supply, but improve crop diversification offering sorghum as an alternative in corn–soybean dominated farming systems.

Studies have been conducted to characterize production environments (Lobell et al., 2013, 2014; Seifert et al., 2018). Although understanding environment challenges is useful to design selection strategies, it should not be confounded with the characterization of the TPE. In the past, simulation models have proved to be effective in assisting the breeding progress (Cooper and Hammer, 1996; Chapman et al., 2000; Hammer et al., 2005; Messina et al., 2009). Cooper and Hammer (1996) compared methods for the analyses of multienvironment trials and the characterization of environments, including the use of crop models. Chapman et al. (2000) used a model for sorghum in Australia as a first step in the TPE identification and characterization of stress patterns due to water-limitation. This approach prevents biased selection due to inadequate sampling of the TPE (Hammer and Jordan, 2009; Messina et al., 2011) and enables weighted selection (Cooper and Podlich, 1999). Since crop growth models are building on a legacy of physiological understanding of plant adaptation, the use of such model enables the breeder to access a physiological interpretation of the G x E basis (Chenu et al., 2009, 2011; Hammer et al., 2010; Cooper et al., 2021). Despite the importance of the crop to the diversity and production agriculture in drought-prone environments, an environmental classification for sorghum in the main Sorghum Belt region of the US is lacking.

This study utilized a dataset from 15 different sites within the US “Sorghum Belt” region spanning from 2015 to 2020 period to calibrate and validate the model, a historical weather record spanning 1984–2020 period to characterize the main water and heat environments, and finally 10 sites-years from 2007 to 2013 to test the model conclusions. The main objectives of this study were to: (i) calibrate and validate APSIM-sorghum crop model to describe the most representative heat and water patterns for the study region sorghum-belt, (ii) identify and test the defined environment clusters with a dataset retrieved from university-driven sorghum hybrid performance trials, and (iii) evaluate the model sensitivity to discriminate environments within a relevant yield-driven classification to assist breeders, agronomists, and farmers for sorghum.

## Materials and Methods

### Model Development: Calibration, Validation, and Definition of Water and Heat Stress Patterns

APSIM-Sorghum model (Hammer et al., 2010), included in the Agricultural Production Systems Simulator software platform (APSIM: Holzworth et al., 2014), was calibrated using data collected from field trials conducted at Riley, Kansas in United States, during the 2018 and 2019 growing seasons. We understand as calibration to the iterative adjustment of model parameters (and in this case, cultivar crop coefficients) until the comparison of the simulated outputs with the experiments observed values presented acceptable model performance. Two commercial hybrids with relative maturities of 70 and 71 (Table 1; Corteva Agriscience, Johnston, IA, United States) were selected as representative materials of the region for this study. Phenology parameterization was conducted using the observed number of expanded leaves throughout the crop growth life cycle, thermal time to anthesis, and thermal time to physiological maturity. Leaf area measurements were used to fit sigmoid curves for total leaf area per plant as a function of thermal time from emergence (Hammer et al., 1993; Hammer and Muchow, 1994) for each genotype. Dry mass accumulation, grain number, grain size, and grain yield data from the field studies were used to derive estimates for each genotype of the coefficient, relating grain number to biomass (Rosenthal et al., 1989; Heiniger et al., 1997). See Supplementary Material for further details related to experimental design and field determinations.

**TABLE 1:** Hybrid description.

Hybrid	Maturity	Relative maturity
**Calibrated hybrids**		
Hybrid 1	Mid-Late	70
Hybrid 2	Mid-Late	71
**Testing hybrids**		
84G62	Mid-Late	72
85G01	Mid	69
85G03	Mid	69
85G46	Mid	68

Model validation was executed using field trials conducted from 2015 to 2020 growing seasons for a total of 15 site-years in the US Sorghum Belt region (Table 2). Validation is defined as the process where the model outcomes (using the coefficients from the calibration) are compared against an independent observed dataset. Data for the validation of days to anthesis was available only in six site-year combinations from 2015 to 2019. The model validation trials included experiments under different management conditions. Details on site characteristics and management practices of each trial are included in Table 2. Soil data was obtained from USDA-SURGO (www.nrcs.usda.gov/wps/portal/nrcs/detail/soils/survey-, visited January 2021), and soil parameters were calculated following Archontoulis et al. (2014). Precipitation and temperature observed data were collected from NOAA weather stations (www.noaa.gov, visited January 2021), considering the nearest weather stations to the experimental site (less than 20 km). Solar radiation was gathered from NASA-POWER project (https://power.larc.nasa.gov/, visited January 2021). Days to anthesis and grain yield was measured and compared with simulations. Observed and simulated value agreement was tested using root mean square error (RMSE: the lower the value the better), normalized root mean square error (NRMSE), and the Nash- Sutcliffe efficiency (NSE, the closer to 0 the value the better, Jin et al., 2010).

**TABLE 2:** Detailed field experiments on sorghum conducted for model parameterization and testing.

Site	Year	Rain	Mean temperature (°C)	Minimum temperature (°C)	Maximum temperature (°C)	SAWC	Thickness (mm)	Irrigation amount (mm)	Planting date	Previous crop	Plant density (plants/ha)	N fertilizer (kg/ha)
Beaver, OK	2016	476	14.7	8.0	21.5	0.338	2030	0	4-June	Corn	42000	27
Beaver, OK	2017	462	13.9	5.8	22.0	0.375	2030	150	14-June	Corn	70000	55
Cloud, KS	2015	763	12.9	7.0	18.8	0.539	2000	0	24-June	Soy	70000	55
Cloud, KS	2017	714	13.1	6.7	19.4	0.550	2000	0	13-June	Sorghum	70000	55
Cloud, KS	2018	664	12.0	5.7	18.2	0.550	2000	0	6-June	Soy	70000	55
Cloud, KS	2019	885	12.4	6.7	18.1	0.739	2000	0	1-July	Soy	70000	55
Cloud, KS	2020	600	13.8	7.5	20.1	0.739	2000	0	4-June	Soy	90000	55
Dallam, TX	2019	205	14.8	7.4	22.3	0.554	1350	150	14-June	Corn	36000	27
Dickinson, KS	2020	756	12.5	5.6	20.2	0.540	990	0	1-June	Soy	90000	55
Finney, KS	2018	545	12.3	4.2	20.3	0.457	2000	150	10-June	Corn	60000	55
Hale, TX	2016	464	16.4	8.7	24.0	0.558	2030	150	26-May	Soy	70000	55
Hockley, TX	2015	690	15.3	7.7	22.8	0.304	2030	0	18-May	Corn	60000	55
Lipscomb, TX	2020	325	17.7	10.4	25.0	0.363	2030	150	26-May	Corn	48000	27
Lubbock, TX	2020	293	15.9	8.3	23.5	0.360	2030	150	28-May	Corn	60000	55
Moore, TX	2018	250	13.9	5.8	21.9	0.492	2030	150	29-June	Corn	38000	27
Riley, KS	2016	859	13.9	8.2	19.5	0.578	1350	0	8-June	Soy	70000	55
Riley, KS	2018	541	13.2	6.2	20.1	0.554	1350	150	7-June	Corn	70000	55
Riley, KS	2019	881	12.7	6.4	19.1	0.554	1350	0	8-June	Wheat	70000	55
Thayer, NE	2016	673	12.2	5.4	18.9	0.820	2000	0	6-June	Wheat	70000	55
Thayer, NE	2017	572	12.0	5.0	19.0	0.820	2000	0	6-June	Wheat	70000	55
Wamego, KS	2020	681	12.5	6.1	18.9	0.485	1930	0	16-June	Soy	10000	55
Wamego, KS	2020	681	12.5	6.1	18.9	0.485	1930	0	16-June	Soy	70000	55
Wamego, KS	2020	681	12.5	6.1	18.9	0.485	1930	0	16-June	Soy	105000	55
Wamego, KS	2020	681	12.5	6.1	18.9	0.485	1930	0	16-June	Soy	10000	82
Wamego, KS	2020	681	12.5	6.1	18.9	0.485	1930	0	16-June	Soy	70000	82
Wamego, KS	2020	681	12.5	6.1	18.9	0.485	1930	0	16-June	Soy	105000	82
Wichita, KS	2016	267	13.9	7.5	20.3	0.586	2000	0	7-June	Wheat	70000	55
Wichita, KS	2017	515	12.6	4.3	20.9	0.586	2000	0	19-June	Wheat	70000	55
Wichita, KS	2019	313	15.3	7.9	22.7	0.575	2030	150	31-May	Sorghum	60000	55

*Experiments involved two sorghum hybrids under a range of N and water regimes over a period of 5 years throughout Kansas (KS) and Texas (TX), conducted at Corteva Agriscience research stations. SAWC stands for soil available water content.*

The characterization of water (WSP) and heat (HSP) stress patterns was achieved by utilizing the same sites from the model validation and retrieving historical weather records from the nearest NOAA weather stations (less than 15 km from the experimental site, Table 2).

Based on the NASS progress report, 50% of the sorghum was planted June 8, an average of the last 10 years (USDA National Agricultural Statistics Service, 2021), and thus to represent the regional management practices, four sowing dates were simulated: May 15, June 1, June 15, and July 1. Moreover, we define two fertilization-plant density combinations: (a) high N-plant density, 138 kg N ha^–1^ and 28 plants m^–2^ and (b) low N-plant density, 69 kg N ha^–1^ and 14 plants m^–2^, reflecting the main sorghum production practices in the area (McHenry, 2016). The initial conditions were set as 50 mg kg^–1^ of NO_3_ and half of the soil water holding capacity. Both calibrated genotypes were used in the simulations for the water and heat characterization. This setup was representative of the yield trends in Kansas, compared with NASS survey data (Supplementary Figure 1A).

Two outputs were required from the APSIM-Sorghum model: (i) the water supply to demand ratio or relative transpiration (RT) index, which limits dry mass accumulation and generates the drought stress impact index (Hammer et al., 2010), and (ii) the heat-stress effect on seed set through the grain temperature factor (GT), which is calculated as the fraction of the sensible window (50°Cd before anthesis and 100°Cd after anthesis), with daily maximum temperatures over 32°C (Singh et al., 2015, 2016). The RT and GT were recorded daily to simulate the time series of water and heat stress for each site × year × management combination, and then averaged every 100°C days from emergence to maturity.

Water and heat outputs were treated independently. The definition of the patterns through clustering was done following Chapman et al. (2000). Within each data base (water and heat), the stress indexes (RT and GT) seasonal trajectories of all the simulations were grouped according to their similarities to define patterns that characterized the whole region. With that purpose, the k-means clustering algorithm was applied to all simulated time series (R Core Team, 2020) to identify the WSP and HSP. To define the number of clusters, NbClust R package (Charrad et al., 2014) was used. Lastly, frequency distributions for both patterns (WSP and HSP) were described for each site.

### Model Testing: Observed vs. Simulated Water and Heat Stress Patterns Comparison

We refer as model testing the assessment of the sensitivity of the environmental classification, WSP and HSP clusters, and their association with observed G x E interaction. For this purpose, an independent dataset retrieved from the Kansas State University Grain Sorghum Yield Performance Tests (agronomy.k-state.edu/services/crop-performance-tests/grain-sorghum, accessed 2021/06) was utilized. This dataset is an extensive collection of yield data spanning from 21 years of data, from 1992 to 2013, with a total of 270 site-year combinations, and with roughly 65 hybrids tested in each site-year. Kansas sorghum tests are located on university-owned research facilities or on privately owned farms, representing the primary growing regions in the state of Kansas. The entry selection and site are voluntary, and not all hybrids are grown at all test sites. Therefore, the dataset is highly unbalanced for genotypes and sites within each year and from year to year. Each plot consisted of two-row plots with length ranging from 20 to 30 feet at the different sites. The experimental design was a randomized incomplete block design with no replications. Grain yields are adjusted to a moisture content of 12.5%. The general management practices included N fertilizer rates of 119 ± 27 kg N ha^–1^, plant density of 17 ± 4 plants/m^–2^, and planting dates ranging from Apr 14 to July 7.

From the dataset described above, subset of hybrids was selected considering only Pioneer hybrids to align with model calibration/validation, using only the site-year combinations where calibrated sorghum hybrids data were available to be included in the field model evaluation. Additionally, a specific sorghum hybrid (or genotype) was included in the dataset only if this material was tested at least in five site-year combinations to build a more representative and robust database. The outcome of this subset was a database with 6 hybrids ranging from 68 to 72 relative maturity (84G62, Hybrid 2, 85G01, 85G03, 85G46, Hybrid 1; Table 1) tested in 10 sites-years from 2007 to 2013 (Supplementary Figure 2A). Using the simulation of Hybrid 1 in each site-year with the respective management of each trial, the time series trajectories for water deficit and heat were accounted for using the procedure described in Section “Model Testing: Observed vs. Simulated Water and Heat Stress Patterns Comparison.” Then each one of these trajectories was classified into one of the previously described WSP and HSP based on the minimum sum of squared differences (Chenu et al., 2011). Variance components were estimated using mixed models implemented in the lme4 R package (Bates et al., 2015).

### Model Sensitivity: Assessment of “Yield-Relevant” Environmental Classification

To enhance the interpretation, each year × site was regrouped according to their differences in yield. This environmental classification comprised four groups: no stress, heat stress, water stress, and both heat and water stress. A mixed model was fitted using the site, and the new environmental classification nested in site as random effects to account for the variance components (Bates et al., 2015). Lastly, a principal component analysis (PCA) was conducted with these four categories, and the hybrid by site matrix gaps were filled with predicted values as detailed by Yan (2013, 2016).

## Results

### Model Development: Calibration, Validation, and Definition of Water and Heat Stress Patterns

**FIGURE 1:**
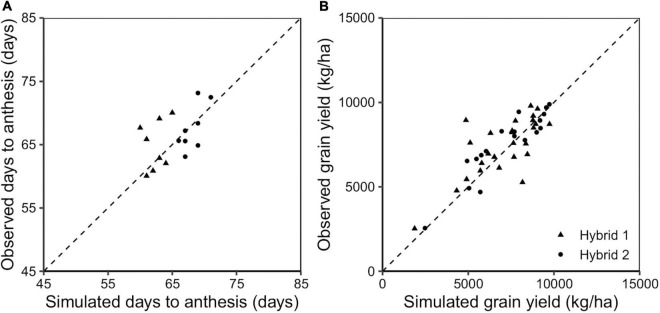
Simulated vs. observed days to anthesis **(A)** and grain yield **(B)** for the hybrids Hybrid 1 (triangles) and Hybrid 2 (circles) for the evaluation testing trials. The slashed line represents the 1:1 line.

The APSIM-Sorghum model was calibrated and validated for two representative genotypes in the Sorghum belt region. Days to anthesis and grain yield were in agreement between observed and predicted values for the calibrated genotypes across sites and years (Figure 1). Differences between simulated and observed days to anthesis were minor (RMSE = 3.6 days; Table 3) and simulated yields were within the experimental standard deviation of observed means (RMSE = 1279 kg/ha). The Nash model efficiency index ranged between 0 and 1, indicating good model performance (Table 3). Larger deviations between simulated and observed data were not strictly linked to poor model performance under low or high yielding values (Figure 1). Pearson correlation values showed a good correlation between observed and predicted values (*r* = 0.80–0.86, Figure 1 and Table 3). A second step was running long-term simulations (from 1984 to 2020) using the model validation sites as representative sites of the sorghum-cropping area of the US Great Plains. The simulated yield matched the variability across years and sites as shown in Supplementary Figures 1A,B.

**TABLE 3:** Measures of agreement between model and measured data.

Performance index	Grain yield	Days to anthesis
Pearson correlation	0.80	0.86
RMSE	1279 kg/ha	3.6 days
NRMSE	62.2	92.2
NSE	0.60	0.092

*RMSE, root mean square error; NRMSE, normalized root mean square error; NSE, Nash model efficiency.*

**FIGURE 2:**
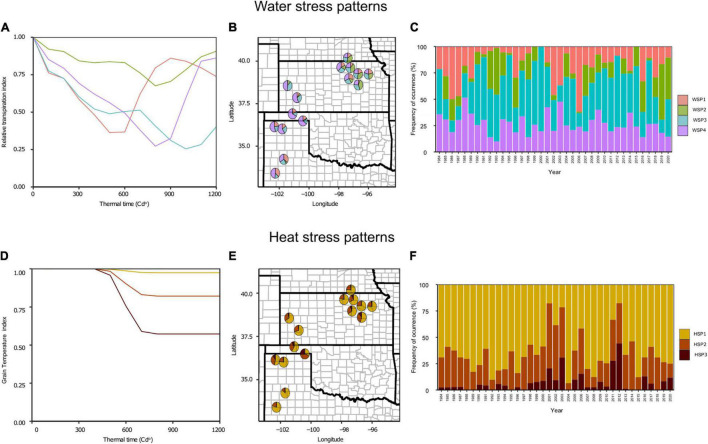
**(A,D)** Relative transpiration (RT) index **(A)** and grain temperature (GT) index **(D)** throughout the crop life cycle expressed in thermal time units for the identified patterns. Water stress patterns (WSP): WSP1, in green is a low water deficit condition; WSP2, in red is a preflowering water deficit condition; WSP3 in blue is a grain filling water deficit condition with no recovery at the ends of the crop life cycle; WSP4 in purple is a grain filling water deficit condition with recovery at the ends of the crop life cycle. Heat stress patterns (HSP): HSP1, in yellow is a low heat stress condition; HSP2, in orange is a moderate heat stress condition; HSP3 in dark red is a severe heat stress condition. Each line represents the index average for the seasons clustered under the same group. The RT and GT values go from 1 to 0, with 1 representing a no stress condition and 0 a complete stress condition. **(B,E)** United States Great plains map with the WSP **(B)** and HSP **(E)** frequencies are represented as pie charts. The pie charts are placed in the trial sites for the model evaluation. **(C,F)** Frequency of the occurrence of the different WSP **(C)** and HSP **(F)** over 36 years of climatic data for the whole studied region.

Four WSP were defined, which accounted for 45% of the total phenotypic variance (Figure 2A). The first stress pattern (WSP1) was characterized by water deficit stress around flowering and later recovery, the second (WSP2) was defined as a low water deficit conditions, the third (WSP3) with water stress patterns gradually increasing from the first third of grain filling and continuing until maturity, and the fourth (WSP4), similar to WSP3, recovering by precipitation events during the end of grain filling. The frequency of occurrence of the WSP1 to WSP4 was 19, 16, 26, and 40%, respectively. However, the region can be subdivided into two distinctive areas: (i) the northeast and (ii) the southwest region (Figure 2B). The northeast area displayed even distribution of WSP (WSP1:17%, WSP2:29%, WSP3:29%, and WSP4:25%), whereas the southwest region was mainly characterized by the WSP4 (WSP1:21%, WSP2:3%, WSP3:23%, and WSP4: 54%). Thus, water stress during the grain filling period was predominant (WS3 + WS4 ca. 75%) in the southwest region.

On the other hand, three HSP accounted for 83% of the variation (Figure 2D). The main difference on these patterns was the intensity of the stress, differentiating a low (HSP1), moderate (HSP2), and a severe (HSP3) level. The low stress condition (HSP1) was the most common pattern for the entire region (67%). In contrast, severe cases of heat stress (HSP3) were scarce (6%). No spatial pattern was apparent from the analysis (Figure 2E). Furthermore, neither WSP nor HSP presented a clear temporal trend (Figures 2C,F).

**FIGURE 3:**
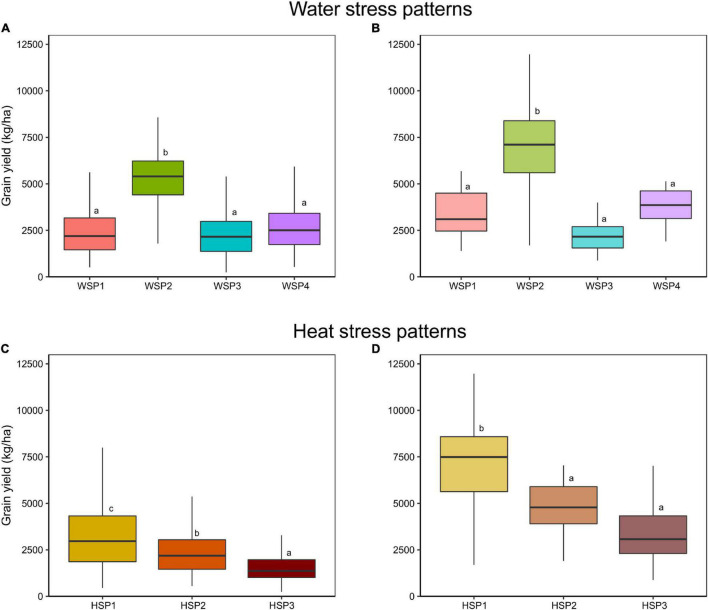
Simulated **(A,C)** and observed **(B,D)** yield distribution of the hybrids Hybrid 1 and Hybrid 2, within each **(A,B)** WSP and **(C,D)** HSP. Different letters inside each graph stand for significant differences in Student’s *t*-test.

Simulated yield was significantly affected by both WSP and HSP, yet the interaction between HSP and WSP was not significant (Supplementary Table 1 and Figures 3A,B). As expected, the stress patterns with the lower intensity (WSP2 and HSP1) presented the highest yields (Supplementary Table 1 and Figures 3A,B). Moreover, the effect of the HSP was related with the degree of stress (HSP1 > HSP2 > HSP3), where the environments classified as HSP3 yielded the lowest of the group. Among WSP, the differential was mostly due to the onset of the water deficit, and therefore the impact on yield is highly dependent on the physiological event that occurs in the crop during the environmental deficit event. WSP3 and WSP1 presented the lowest yields, related to the effect of the stress around the flowering period (WSP2 > WSP4 > WSP1 > WSP3). WSP and HSP description for each site is available in Supplementary Table 2.

### Model Testing: Observed vs. Simulated Water and Heat Stress Patterns Comparison

The dataset from calibrated hybrids 1 and 2 was in agreement (Supplementary Figure 2B, RMSE = 1,353 kg/ha) with the independent dataset provided by KSU grain sorghum yield performance tests. The calibrated genotype Hybrid 1 yields did not differ from the rest of the Corteva hybrids (Supplementary Figure 2C) across, and was therefore considered as a representative hybrid for the following simulations. Furthermore, the independent dataset explored a wide range of weather conditions (Supplementary Figure 2D).

The comparison between the simulated vs. field datasets corroborated the defined patterns for grain yield. Observed and simulated grain yield portrayed a similar trend for both WSP and HSP (Figures 3A,C vs. Figures 3B,D). The WSP2 presented higher yields relative to other patterns, acting as the non-stress scenario. Regarding HSP, the observed classification showed significant differences according to the intensity of the stress, with greater heat stress (HSP2, HSP3) associated with lower grain yields.

### Model Sensitivity: Assessment of “Yield-Relevant” Environmental Classification

**FIGURE 4:**
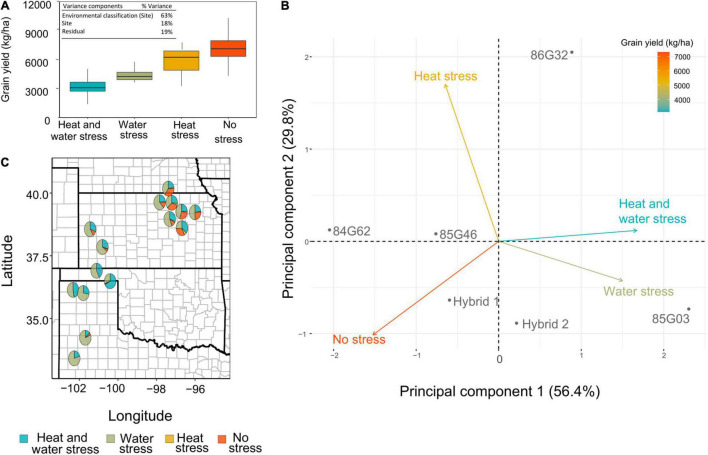
**(A)** Observed yield distribution for the four environmental classification groups. (**A**, inset) Variance components for observed grain yield in field experiments are expressed as percentage of the total variance explained by each effect. **(B)** Principal component analysis for observed yield. The points represent the six evaluated hybrids and the vector the four environmental categories. **(C)** United States Great plains map with the environment category group (ECG) frequencies represented as pie charts. The pie charts are placed in the trial sites for the model evaluation.

The abiotic environmental stressors were further reclassified into a new environment category group (ECG) according to the clusters’ significant impact on yield (Figures 3B,D): No stress (HSP1; WSP2), heat stress (HSP2 and HSP3; WSP2), water stress (HSP1; WSP1, WSP3, and WSP4), and combined heat and water stress (HSP2 and HSP3; WSP1, WSP3, and WSP4). This approach provides a simplified characterization of the interactions that would be easier to adopt by breeders, agronomists, and farmers. This classification discriminated stress trajectories for all site-years more clearly (Figure 4A), with “No stress” clustered environments presenting the highest yields and the “Heat and Water” stress cluster resulting in the lowest yields. The mixed model result showed that the ECG can account for more than 60% of the variation of the observed yield variance (Figure 4A, inset).

Furthermore, a PCA analysis explored the interactions among the ECG and accounted for 86% of the observed grain yield with the first two PCs (Figure 4B). From the PCA, the “No stress” ECG and “Heat and Water” stress ECG displayed a negative correlation (angle close to 180°). The water stress ECG was correlated with heat and water stress ECG, as reflected in the biplot with a reduced angle. In contrast, heat stress ECG presented an angle close to 90°, with both heat and water and no stress ECGs, which clearly points out to the lack of correlation between these environments. The size of all vectors was similar, indicating that the ECG presented similar weights to explain the observed yield. The PC 1 assisted in discriminating environments based on yield, with the highest yield environments resulting in lower values of PC 1.

This new set of ECGs was applied to the long-term weather simulations and a new environmental characterization was developed for relevant sites within the US Sorghum Belt region (Figure 4C). In summary, high proportion of environments with “No Stress” were concentrated toward the east of the region, heat stress was jointly present with water stress, and heat only was less frequent but more evident, again toward the east of the region. Water stress was the most relevant indicator for the entire sorghum region.

## Discussion

The environmental characterization presented in this study is a first step on the path to: (i) improve crop adaptation to the environment. It nurtures the breeder knowledge regarding the main sources of yield variability in the region, exploring G x E interactions while also covering the entire geographical space. (ii) Enhance genetic gains in sorghum breeding, and this is accomplished through improving the criteria for selecting breeding locations with objective measurements of environmental representativeness. Hence allowing a more efficient use of the breeding program resources and opening the possibility of defining subprograms focused on specific environments. Lastly, (iii) increase the attainable yield at the farmer’s production field. Although this approach is showing promising results, it is worth highlighting that additional detailed field data (e.g., crop phenology, yield, and weather environments) will be needed to accurately test the crop performance impacts of all-stress pattern combinations.

High temperatures can severely impact sorghum productivity (Prasad et al., 2006). The APSIM-sorghum model was able to capture significant high temperature effects on yield, mostly under contrasting heat levels, yet it has limitations to accurately represent the overall impact of the heat stress (Jin et al., 2016). The temperature impact in the model is mainly reflected as the grain set’s response to heat extremes (Singh et al., 2015, 2016), although the parameterization of this mechanistic process has not been extensively tested. In addition, the current temperature approach in the model neither takes into consideration the effect of night temperatures on grain set and grain formation (Prasad et al., 2006), nor the effect of the high temperatures on grain size during grain filling (Prasad et al., 2015, 2017). More recently, Sunoj et al. (2020) reported that an effect of diurnal temperature and night respiration variation for sorghum should be considered as critical factors to screen for hybrids with greater heat tolerance. Despite the low frequency of high temperature in the region (severe stress was less than 5%), high day and night-time temperatures are expected to change over the next decades due to global warming, and heat could become a major limiting factor to restrict growth, development, and productivity in sorghum (Prasad et al., 2006, 2015; Lobell et al., 2013; Singh et al., 2014).

Sorghum is characterized for improved drought tolerance, which is reflected in the main changes in root architecture and water capture (Singh et al., 2010). In addition, under drought, positive relationship was reported for yield and grain number (Mutava et al., 2011). Although sorghum tolerance to drought is broadly recognized as a relevant trait (Blum, 1974; Blum and Arkin, 1984; Blum, 2009), water availability was encountered as the main yield-limiting factor for this region. The model-based approach to defined water patterns can be mainly criticized in the relatively constant ability of the plant to resume its growth after a stress is relieved. This scenario seems more reasonable when the crop is exposed to low intensity of drought for long periods (Ben Haj Salah and Tardieu, 1997), but not to a severe stress when this condition can cause damage at the cellular level losing the ability of the plant to fully recover from this stressor (Prasad et al., 2019). Nevertheless, this approach has been widely tested and its conclusions validated in sorghum, maize, mungbean, wheat, chickpea, rice, and pea (Chenu et al., 2011, 2013; Sadras et al., 2012; Chauhan et al., 2013; Kholová et al., 2013; Chauhan and Rachaputi, 2014; Cooper et al., 2014; Hammer et al., 2014; Harrison et al., 2014; Heinemann et al., 2015, 2019; Lobell et al., 2015; Seyoum et al., 2017, 2018; Crespo-Herrera et al., 2021). Using the same method in maize (*Zea mays* L.) in the US Corn Belt, Cooper et al. (2014) identified relevant water patterns and characterized their spatial distribution for the region. For both crops (sorghum and maize) any of the identified temporal patterns of water balance can occur in any location–year combination, albeit with different expected frequencies of occurrence and defined similar low water stress spatial distribution (increasing from west to east).

In environments that are prone to low predictability of the prevailing water pattern (as shown in eastern KS), defining *a priori* the traits and magnitude of genetic variation for grain yield that will be revealed at each site–year combination is a very difficult task (Messina et al., 2011; Cooper et al., 2014). This highlights the relevance of identifying these patterns and the associated genetic variation at the level of the TPE (Chapman et al., 2000; Chenu et al., 2011). Thus, describing the TPE through characterizing the most relevant patterns could assist breeders to (i) interpret and account for the effects of G x E, (ii) weight genotype performance by the representativeness within a TPE, (iii) choose trial sites and design experiments to select new genotypes with superior performance for this environments, (iv) test specific physiological and breeding hypotheses, such as those associated with identifying of key adaptive traits. Furthermore, environments, such as the ones from western KS, with low prevalence of optimal conditions involving low use of inputs, suggests that breeding program would benefit through direct selection in non-optimal conditions, even if genetic gains are lower under such conditions (Heinemann et al., 2015).

Heat and drought are highly related environmental stressors: higher temperatures increase the vapor pressure deficit, which could drive drought stress (Prasad et al., 2019). However, it is not uncommon to have one without the other (Sadras et al., 2012). Thus, it is important to target them separately as the traits that lead to tolerance in one may differ from the other. For instance, traits related with the root architecture (Singh et al., 2010) or the stay green (Borrell et al., 2014), will impact mainly the drought tolerance, whereas pollen viability and seed-set percentage under heat stress (Nguyen et al., 2013) or higher cardinal temperatures (Djanaguiraman and Prasad, 2014) will have a bigger impact on heat-stress tolerance. Therefore, the candidate germplasm tested and the selection criteria will also differ upon different evaluating conditions (i.e., eastern KS environments will benefit more broadly from adapted genotypes while western KS environments will favor germplasm adapted to grain filling water-stress tolerance).

From the farmer’s perspective, uncertainty is a central aspect of new technology adoption, especially in the context of agriculture where the relevance and suitability of a new technology for a specific farm depend on both the farmer’s background knowledge (Mase et al., 2017; Huffman, 2020) and on environmental conditions. Furthermore, there is an uncertainty on how best to use a technology, in particular if it is used in combination with other management practices (i.e., fertilizers × plant densities × planting dates). This is a clear research knowledge gap that is not addressed in this article. We should consider how to design crop improvement strategies that can explore the diverse opportunities within the potential space of G x E x M possibilities to achieve sustainable improvements in crop productivity (Braun et al., 2010; Chapman et al., 2012; Snowdon et al., 2021). Targeting G x M technology combinations creates opportunities to exploit G x M interactions within the classified environments. For example, the characterization of the TPE could be applied to improve the use of the available water. Selecting early flowering hybrids and low tillering genotypes could help escaping late-season stress and using water resources in a more efficient way (Hammer et al., 2014). For the western Sorghum Belt, more relevant grain filling water stress conditions and limited irrigation should be focused on timing water needs with the plant demand, which ultimately will increase the profitability of the farming operation (Araya et al., 2021). In contrast, the eastern region presented a high frequency of low stress WSP, representing a challenge on how to pursuit higher yielding when the current available hybrids might be limited by potential (Cooper et al., 2014; Broeckelman et al., 2015; Broeckelman, 2016).

Even though there are limitations to this environmental classification approach, future research could help improving these models by: (i) exploration in the use of Bayesian models to account for uncertainties on the estimation of environments (spatial–temporal scale) (Liverani et al., 2015); (ii) expanding the geographical footprint and using crop growth modeling tools to address the effect of future climate on the current classification and changes in frequency of environment types (Harrison et al., 2014; Lobell et al., 2015; Hammer et al., 2020); (iii) expanding the efforts on collecting detailed physiological evaluations of more and diverse sorghum germplasm to better assess genetic variation in the models and lastly, (iv) the method has limitations of the space of G x E x M combinations and inference related to technology adoption, including the influence of management on the environment types and their frequency of occurrence (Chapman et al., 2012; Hammer et al., 2014, 2020; Lobell et al., 2015) is a clear step forward. Genotype by environment by management interactions underpin many aspects of crop improvement and thus, the design of new strategies is still relevant to explore the potential space of G × E × M possibilities for each TPE.

## Conclusion

Relevant grain sorghum environments were classified for the US Great Plains region using a classical approach integrating relevant soil, weather, and field data. Four outcomes are worth highlighting from this study: (i) the defined patterns assisted in explaining the basis for the observed G x E interaction for yield, (ii) knowledge of the spatial and temporal distribution of the most frequent patterns can help defining sites for evaluation trials, design of breeding programs, future target traits, and exploring innovations linked to crop management, (iii) the validation of the patterns *via* a sensitivity analysis with an independent dataset is a novel approach that should be included in future environtyping framework, and (iv) clustering low frequency patterns to explore the most relevant environments demonstrated to be a valid strategy to create a classification easier to be applied by breeders, agronomists, and farmers, without introducing any noticeable trade-off.

There is still considerable scope to improve the environmental assessment involving the definition of uncertainties, accounting for the management component, and the influence of future climate change. Improving environmental classifications, which hinges effective evaluation of genotype and management practices, is a prerequisite to deal with the future projections of food demand, and to constraint the threatens on food security.

## Data Availability Statement

The raw data supporting the conclusions of this article will be made available by the authors, without undue reservation.

## Author Contributions

AC and IC conceived the presented idea. AC carried out the simulations. CM, GM, and LM verified the analytical methods. PD led field studies for calibration data. JL led field studies for testing data. AC wrote the first draft of the manuscript with support from IC. All authors discussed the results and contributed to the final manuscript.

## Conflict of Interest

LM was employed by Corteva Agriscience. The remaining authors declare that the research was conducted in the absence of any commercial or financial relationships that could be construed as a potential conflict of interest.

## Publisher’s Note

All claims expressed in this article are solely those of the authors and do not necessarily represent those of their affiliated organizations, or those of the publisher, the editors and the reviewers. Any product that may be evaluated in this article, or claim that may be made by its manufacturer, is not guaranteed or endorsed by the publisher.

## Abbreviations

WSP: water stress patterns
HSP: heat stress patterns
RT: relative transpiration index
GT: grain temperature index
ECG: environment category group.
