# Supplementary material for: Environment Characterization in Sorghum (Sorghum bicolor L.) by Modeling Water-Deficit and Heat Patterns in the Great Plains Region, United States
Source: Front Plant Sci. 2022 Mar 3;13:768610. doi: 10.3389/fpls.2022.768610 (PMC8929132; doi:10.3389/fpls.2022.768610)
Supplement: Supplementary file 1 [file Data_Sheet_1.docx]

**Supplementary data D1**

*Experimental conditions*

Field studies were conducted at Corteva Agriscience research station in Riley, Kansas (KS), USA, during the 2018 and 2019 growing seasons.

Across all sites, the experimental design was a randomized complete block design (RCBD), with 2 sorghum hybrids (85Y40 and 84P80) and three replications. For the 2018 growing season, the plot size was 2-row plots with 5.3 m long and 0.76 m row spacing in all locations, the planting date was 6/7/2018 and had limited irrigation. For the 2019 season, the plot size was 8-row plots with 5.3 m long and 0.76 m row spacing, the planting date was 6/8/2019, and was managed as a dryland system. The trials were utilized to obtain data on grain yield, and detailed physiological descriptors of grain yield formation. In the study, no fertile tillers were considered in the data collection, and data analysis due to the high plant density (heads number = plant number. The plant density for both growing seasons was the same (173.000 plans/ha).

*Measurements and laboratory analysis*

Aboveground plant biomass was measured at flowering and at harvest time during the 2018 season by collecting four consecutive plants per plot due to the small plot size. For the 2019 season, biomass samples were collected at three times during the vegetative phase (5 fully expanded leaves, 8-9 fully expanded leaves, and 15 days before flowering) and three times during the reproductive phase (flowering, 15 days after flowering, maturity) in a 0.7 m2 harvest area. During the vegetative stages, plants were partitioned into leaves (leaf blades) and stem (stem + leaf sheaths). During reproductive stages, plants were partitioned into leaves, stem, and panicle (plus grain). Dry weight was obtained after drying plant fractions in an air-forced oven at 65°C until constant weight. Phenology was recorded daily for tagged individual plants before flowering and during the reproductive period.

Leaf area was measure from the same plants collected for biomass, leaf size was determined using the Li-Cor 3000 equipment to determine the total per-plant leaf area (in cm2).

Grain yield was obtained from a plot combine, harvesting two rows per plot (approximately 8.17 m-2) and adjusted to a standard 150 g kg-1 moisture. Grain number per unit of land area was calculated as the ratio between total grain yield and individual grain weight.

Calibrated parameters

| Parameter | Hybrid 1 | Hybrid 2 |
| --- | --- | --- |
|  |  |  |
| photoperiod_slope | 0 | 0 |
| tt_endjuv_to_init | 180 | 208 |
| tt_flag_to_flower | 168 | 193 |
| tt_flower_to_maturity | 705 | 685 |
|  |  |  |
| main_stem_coef | 2.94 | 3.06 |
| tpla_prod_coef | 0.009 | 0.008 |
|  |  |  |
| dm_per_seed | 0.0008 | 0.0011 |
| maxGFRate | 0.04 | 0.05 |
|  |  |  |

**Supplementary tables**

**Table S1.** Simulated grain yield means (kg/ha) for each water stress pattern (WSP) and heat stress pattern (HSP), for all the sites and historical series. Variance components are expressed as a percentage of the total variance explained by the effects. Significance differences are indicated with symbols: 0 ‘***’ 0.001 ‘**’ 0.01 ‘*’ 0.05 ‘.’ 0.1 ‘ ’ 1 . Values within different letters are significantly different at P < 0.05.

| Grain yield  (kg/ha) | | | | | | |
| --- | --- | --- | --- | --- | --- | --- |
| WSP | |  |  | HSP | |  |
|  |  |  |  |  |  |  |
| HSP2 | 5430 | a |  | HSP1 | 3243 | a |
| HSP4 | 2669 | b |  | HSP2 | 2387 | b |
| HSP1 | 2436 | c |  | HSP3 | 1551 | c |
| HSP3 | 2265 | d |  |  |  |  |
|  |  |  |  |  |  |  |
| % Variance | |  |  |  |  |  |
| WSP |  | 47 | | *** |  |  |
| HSP |  | 3 | | *** |  |  |
| WSP x HSP | | 0 | |  |  |  |
|  |  |  |  |  |  |  |
| Signif. codes: 0 ‘***’ 0.001 ‘**’ 0.01 ‘*’ 0.05 ‘.’ 0.1 ‘ ’ 1 | | | | | | |

**Table S2.** Relative transpiration index (RT) and grain temperature index (GT), general values description for each site and WSP/HSP respectively.

| Site | HSP | Mean | Max | Min | SD | WSP | Mean | Max | Min | SD |
| --- | --- | --- | --- | --- | --- | --- | --- | --- | --- | --- |
|  |  |  |  |  |  |  |  |  |  |  |
| Cloud, KS | 1 | 0.89 | 1.00 | 0.70 | 0.10 | 1 | 0.71 | 1.00 | 0.11 | 0.28 |
| Cloud, KS | 2 | 0.99 | 1.00 | 0.89 | 0.02 | 2 | 0.84 | 1.00 | 0.21 | 0.19 |
| Cloud, KS | 3 | 0.78 | 1.00 | 0.58 | 0.18 | 3 | 0.56 | 1.00 | 0.01 | 0.30 |
| Cloud, KS |  |  |  |  |  | 4 | 0.65 | 1.00 | 0.05 | 0.29 |
| Dallam, TX | 1 | 0.89 | 1.00 | 0.72 | 0.10 | 1 | 0.61 | 1.00 | 0.07 | 0.28 |
| Dallam, TX | 2 | 0.98 | 1.00 | 0.90 | 0.03 | 2 | 0.68 | 0.93 | 0.38 | 0.18 |
| Dallam, TX | 3 | 0.76 | 1.00 | 0.44 | 0.21 | 3 | 0.48 | 1.00 | 0.05 | 0.25 |
| Dallam, TX |  |  |  |  |  | 4 | 0.56 | 1.00 | 0.08 | 0.27 |
| Finney, KS | 1 | 0.90 | 1.00 | 0.71 | 0.09 | 1 | 0.66 | 1.00 | 0.17 | 0.26 |
| Finney, KS | 2 | 0.98 | 1.00 | 0.91 | 0.03 | 2 | 0.74 | 1.00 | 0.24 | 0.20 |
| Finney, KS | 3 | 0.81 | 1.00 | 0.64 | 0.15 | 3 | 0.54 | 1.00 | 0.01 | 0.31 |
| Finney, KS |  |  |  |  |  | 4 | 0.64 | 1.00 | 0.03 | 0.29 |
| Hale, TX | 1 | 0.90 | 1.00 | 0.72 | 0.09 | 1 | 0.69 | 1.00 | 0.08 | 0.28 |
| Hale, TX | 2 | 0.99 | 1.00 | 0.90 | 0.02 | 2 | 0.79 | 1.00 | 0.13 | 0.21 |
| Hale, TX | 3 | 0.79 | 1.00 | 0.56 | 0.17 | 3 | 0.52 | 1.00 | 0.02 | 0.28 |
| Hale, TX |  |  |  |  |  | 4 | 0.63 | 1.00 | 0.03 | 0.29 |
| Dickinson, KS | 1 | 0.90 | 1.00 | 0.71 | 0.09 | 1 | 0.70 | 1.00 | 0.10 | 0.28 |
| Dickinson, KS | 2 | 0.98 | 1.00 | 0.89 | 0.03 | 2 | 0.84 | 1.00 | 0.14 | 0.20 |
| Dickinson, KS | 3 | 0.79 | 1.00 | 0.40 | 0.19 | 3 | 0.56 | 1.00 | 0.01 | 0.31 |
| Dickinson, KS |  |  |  |  |  | 4 | 0.66 | 1.00 | 0.05 | 0.29 |
| Wamego, KS | 1 | 0.90 | 1.00 | 0.71 | 0.09 | 1 | 0.73 | 1.00 | 0.11 | 0.28 |
| Wamego, KS | 2 | 0.99 | 1.00 | 0.90 | 0.02 | 2 | 0.83 | 1.00 | 0.12 | 0.20 |
| Wamego, KS | 3 | 0.80 | 1.00 | 0.51 | 0.18 | 3 | 0.57 | 1.00 | 0.02 | 0.31 |
| Wamego, KS |  |  |  |  |  | 4 | 0.66 | 1.00 | 0.05 | 0.29 |
| Moore, TX | 1 | 0.88 | 1.00 | 0.70 | 0.10 | 1 | 0.74 | 1.00 | 0.13 | 0.27 |
| Moore, TX | 2 | 0.99 | 1.00 | 0.88 | 0.02 | 2 | 0.84 | 1.00 | 0.07 | 0.21 |
| Moore, TX | 3 | 0.77 | 1.00 | 0.43 | 0.19 | 3 | 0.60 | 1.00 | 0.04 | 0.31 |
| Moore, TX |  |  |  |  |  | 4 | 0.67 | 1.00 | 0.05 | 0.29 |
| Thayer, NE | 1 | 0.91 | 1.00 | 0.72 | 0.08 | 1 | 0.75 | 1.00 | 0.18 | 0.25 |
| Thayer, NE | 2 | 0.99 | 1.00 | 0.90 | 0.02 | 2 | 0.86 | 1.00 | 0.15 | 0.20 |
| Thayer, NE | 3 | 0.79 | 1.00 | 0.60 | 0.18 | 3 | 0.60 | 1.00 | 0.03 | 0.31 |
| Thayer, NE |  |  |  |  |  | 4 | 0.68 | 1.00 | 0.03 | 0.29 |
| Lubbock, TX | 1 | 0.91 | 1.00 | 0.73 | 0.08 | 1 | 0.74 | 1.00 | 0.15 | 0.24 |
| Lubbock, TX | 2 | 0.99 | 1.00 | 0.90 | 0.02 | 2 | 0.85 | 1.00 | 0.12 | 0.19 |
| Lubbock, TX | 3 | 0.79 | 1.00 | 0.60 | 0.18 | 3 | 0.57 | 1.00 | 0.04 | 0.29 |
| Lubbock, TX |  |  |  |  |  | 4 | 0.66 | 1.00 | 0.04 | 0.28 |
| Moore, TX | 1 | 0.89 | 1.00 | 0.71 | 0.10 | 1 | 0.66 | 1.00 | 0.14 | 0.28 |
| Moore, TX | 2 | 0.98 | 1.00 | 0.90 | 0.03 | 2 |  |  |  |  |
| Moore, TX | 3 | 0.79 | 1.00 | 0.57 | 0.18 | 3 | 0.51 | 1.00 | 0.03 | 0.28 |
| Moore, TX |  |  |  |  |  | 4 | 0.57 | 1.00 | 0.06 | 0.28 |
| Hockley, TX | 1 | 0.90 | 1.00 | 0.71 | 0.09 | 1 | 0.63 | 1.00 | 0.07 | 0.29 |
| Hockley, TX | 2 | 0.99 | 1.00 | 0.90 | 0.02 | 2 |  |  |  |  |
| Hockley, TX | 3 | 0.80 | 1.00 | 0.66 | 0.16 | 3 | 0.45 | 1.00 | 0.02 | 0.27 |
| Hockley, TX |  |  |  |  |  | 4 | 0.55 | 1.00 | 0.03 | 0.28 |
| Lipscomb, TX | 1 | 0.89 | 1.00 | 0.67 | 0.10 | 1 | 0.58 | 1.00 | 0.12 | 0.23 |
| Lipscomb, TX | 2 | 0.98 | 1.00 | 0.90 | 0.03 | 2 | 0.72 | 1.00 | 0.22 | 0.22 |
| Lipscomb, TX | 3 | 0.72 | 1.00 | 0.15 | 0.25 | 3 | 0.46 | 1.00 | 0.04 | 0.23 |
| Lipscomb, TX |  |  |  |  |  | 4 | 0.56 | 1.00 | 0.09 | 0.26 |
| Wichita, KS | 1 | 0.91 | 1.00 | 0.71 | 0.09 | 1 | 0.64 | 1.00 | 0.12 | 0.30 |
| Wichita, KS | 2 | 0.98 | 1.00 | 0.89 | 0.03 | 2 | 0.80 | 1.00 | 0.22 | 0.20 |
| Wichita, KS | 3 | 0.81 | 1.00 | 0.55 | 0.16 | 3 | 0.54 | 1.00 | 0.02 | 0.30 |
| Wichita, KS |  |  |  |  |  | 4 | 0.64 | 1.00 | 0.06 | 0.29 |
| Beaver, OK | 1 | 0.90 | 1.00 | 0.70 | 0.09 | 1 | 0.64 | 1.00 | 0.10 | 0.29 |
| Beaver, OK | 2 | 0.98 | 1.00 | 0.89 | 0.03 | 2 |  |  |  |  |
| Beaver, OK | 3 | 0.78 | 1.00 | 0.51 | 0.19 | 3 | 0.45 | 1.00 | 0.02 | 0.27 |
| Beaver, OK |  |  |  |  |  | 4 | 0.54 | 1.00 | 0.06 | 0.26 |
| Riley, KS | 1 | 0.89 | 1.00 | 0.69 | 0.10 | 1 | 0.72 | 1.00 | 0.14 | 0.22 |
| Riley, KS | 2 | 0.99 | 1.00 | 0.90 | 0.03 | 2 | 0.80 | 1.00 | 0.19 | 0.19 |
| Riley, KS | 3 | 0.76 | 1.00 | 0.39 | 0.21 | 3 | 0.60 | 1.00 | 0.08 | 0.26 |
| Riley, KS |  |  |  |  |  | 4 | 0.65 | 1.00 | 0.08 | 0.27 |
|  |  |  |  |  |  |  |  |  |  |  |

**Supplementary Figures**

**Figure S1**. Boxplots representing grain yield throughout (A) years and (B) sites. Red points in (A) represent the average grain yield for Kansas State reported by NASS (USDA, 2021).

**Figure S2.** (A) Number of times each hybrid is present in each site. (B) Simulated versus observed grain yield for the hybrids Hybrid 1 (triangles) and Hybrid 2 (circles) for Kansas State University sorghum hybrid yield performance trials. The slashed line represents the 1:1 line. (C) Observed yield distribution for each site. The point in each boxplot represent the mean of the simulated Hybrid 1 for the simulated trials in each site. The inset is the ANOVA comparing the observed hybrids with the simulated Hybrid 1. (D) Accumulative rain and mean temperature for the selected Kansas State University sorghum hybrid yield performance trials. The different colors stand for the sites using the same color reference as (C).
